# The presence of T cell epitopes is important for induction of antibody responses against antigens directed to DEC205^+^ dendritic cells

**DOI:** 10.1038/srep39250

**Published:** 2016-12-21

**Authors:** Kelly N. S. Amorim, Eline V. Rampazo, Renan Antonialli, Marcio M. Yamamoto, Mauricio M. Rodrigues, Irene S. Soares, Silvia B. Boscardin

**Affiliations:** 1 Laboratory of Antigen Targeting to Dendritic Cells, Department of Parasitology, Institute of Biomedical Sciences, University of São Paulo, São Paulo, 05508-000, Brazil; 2 CTCMol, Federal University of São Paulo, São Paulo, 04044-010, Brazil; 3 National Institute for Science and Technology in Vaccines, Belo Horizonte, 31270-910, Brazil; 4 Department of Clinical and Toxicological Analysis, School of Pharmaceutical Sciences, University of São Paulo, São Paulo, 05508-900, Brazil

## Abstract

*In vivo* antigen targeting to dendritic cells (DCs) has been used as a way to improve immune responses. Targeting is accomplished with the use of monoclonal antibodies (mAbs) to receptors present on the DC surface fused with the antigen of interest. An anti-DEC205 mAb has been successfully used to target antigens to the DEC205^+^CD8α^+^ DC subset. The administration of low doses of the hybrid mAb together with DC maturation stimuli is able to activate specific T cells and induce production of high antibody titres for a number of different antigens. However, it is still not known if this approach would work with any fused protein. Here we genetically fused the αDEC205 mAb with two fragments (42-kDa and 19-kDa) derived from the ~200 kDa *Plasmodium vivax* merozoite surface protein 1 (MSP1), known as MSP1_42_ and MSP1_19_, respectively. The administration of two doses of αDEC-MSP1_42_, but not of αDEC-MSP1_19_ mAb, together with an adjuvant to two mouse strains induced high anti-MSP1_19_ antibody titres that were dependent on CD4^+^ T cells elicited by peptides present in the MSP1_33_ sequence, indicating that the presence of T cell epitopes in antigens targeted to DEC205^+^ DCs increases antibody responses.

DCs are an important bridge between innate and adaptive immune responses. They are able to sense infection and inflammation, and efficiently present pathogen-derived epitopes to T cells^1^. Once activated, T cells produce cytokines and can help activate antibody producing B cells. In addition, DCs are also able to directly activate B cells to mature and produce high affinity antibodies^2^.

Because of their central role in the induction of immunity, manipulation of DCs is an interesting strategy to induce adaptive immune responses. Among these strategies, the use of mAbs to directly target DCs *in situ* has been tested with success in different models^3^,^4^,^5^,^6^,^7^. This is accomplished by the use of mAbs that target different DC surface receptors fused to antigens derived from pathogens, cancer cells, etc.^8^. The C-type lectin DEC205 (CD205) has been used with success to induce both cellular and humoral immune responses^5^,^6^. Despite its expression by other cell types as B cells and epithelial cells^9^,^10^, the DEC205 expression in DCs is responsible for T cell activation when the antigen is targeted *in vivo* through a hybrid αDEC205 mAb^11^,^12^. The use of a DC maturation stimulus together with the hybrid αDEC205 mAb induces long lasting T cell immunity that can even lead to protection in some mouse models of infection^13^,^14^. In addition, the induction of specific antibodies against the targeted antigen has also been observed^3^,^5^.

In summary, there is extensive data in the literature showing that antigen targeting to DCs through the DEC205 receptor elicits CD4^+^ and CD8^+^ T cell activation as well as antibody responses when the hybrid mAb is administered in the presence of a DC maturation stimulus such as αCD40, polyriboinosinic: polyribocytidylic acid (poly (I:C)) or CpG oligodeoxynucleotides^3^,^5^,^6^,^13^,^15^. Among the many antigens delivered to the DEC205^+^ DC subset we can cite the model antigen ovalbumin^13^,^16^,^17^, the tumor antigens survivin^18^, HER2/neu^19^, NY-ESO-1^20^ and melanoma TRP2^21^, and different pathogen-derived antigens such as HIV gag^6^,^7^,^15^, *Yersinia pestis* LcrV^22^,^23^, and *Plasmodium yoelii* CSP^5^,^24^. In all cases, strong CD4^+^ T cell responses were obtained against previously described peptides or against peptides derived from overlapping peptide libraries. CD8^+^ T cell activation was also detected when αDEC205 mAb was fused to ovalbumin, NY-ESO-1, TRP2, HIV gag, or CSP, especially when the CD8^+^ T cells were purified and re-stimulated with single peptides^5^,^6^,^7^,^13^,^21^. However, in some cases, the activation of these cells was not detected^18^,^23^. Taken together, these results indicated that all these antigens possessed antigenic epitopes recognized by the immune system.

Although much has been published with the use of different proteins, the choice of the antigen has not been fully explored. Would any antigen be able to induce strong T cell and antibody responses if targeted to the DEC205^+^ DC subpopulation? To start addressing this question, we fused the αDEC205 mAb with two fragments of the merozoite surface protein 1 (MSP1) derived from *Plasmodium vivax*, the most prevalent species that causes human malaria. MSP1 is expressed during the erythrocytic phase of *Plasmodium* life cycle and participates in parasite invasion^25^. It is expressed as an ~200 kDa precursor on the surface of the merozoite, and undergoes successive proteolytic cleavages generating a 42-kDa fragment (MSP1_42_) that is further cleaved into two products: a soluble 33-kDa fragment (MSP1_33_) that corresponds to the N-terminal region of MSP1_42_ and is shed from the free merozoite surface^26^, and a membrane-bound 19-kDa C-terminal fragment (MSP1_19_), which is the only MSP1 fragment carried with the invading merozoite into the new red blood cell^27^.

Infection with *Plasmodium sp.* leads to the induction of antibodies that bind mainly to the MSP1_19_ protein^28^,^29^,^30^ while MSP1_42_ is thought to contain T cell epitopes^31^ that help B cells to produce anti-MSP1_19_ antibodies^32^,^33^. Antibodies and CD4^+^ T cells directed to MSP1 were shown to be associated with protection against malaria in mice^33^,^34^,^35^ and humans^36^.

To study the differences in terms of antibody induction and T cell activation in the context of DEC205^+^ DC targeting, we delivered MSP1_19_ or MSP1_42_ proteins to this subset through two hybrid mAbs, αDEC-MSP1_19_ and αDEC-MSP1_42_. Analysis of the immune response induced by immunization with the two hybrid mAbs in the presence of poly (I:C) showed that T cell epitopes are indeed present in the MSP1_33_ portion of the molecule and that induction of high titres of anti-MSP1_19_ antibodies is obtained mainly when MSP1_42_ is targeted to the DEC205^+^ DC population.

## Results

### The hybrid αDEC mAbs containing MSP1_19_ or MSP1_42_ fused proteins were successfully produced and capable of binding to the DEC205 receptor

**Figure 1:**
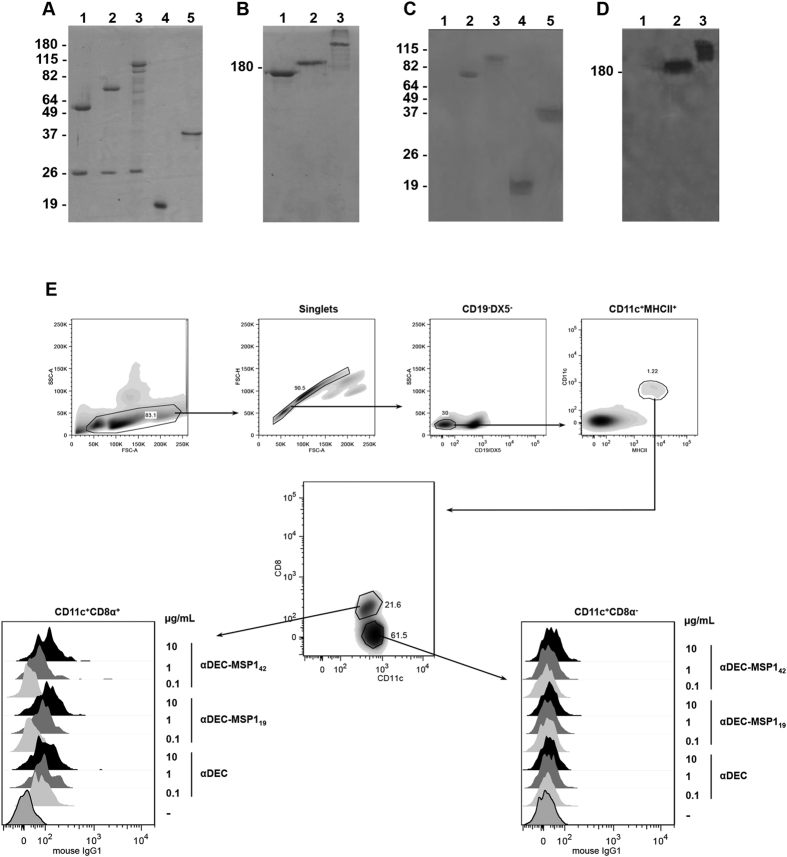
The hybrid αDEC antibodies fused to *P. vivax* MSP1_19_ and MSP1_42_ were successfully produced and recognized by serum from a *P. vivax* infected patient, and retained their ability to bind to DCs expressing the DEC205 receptor. Approximately 1 μg of each hybrid mAb or recombinant protein was separated under reduced (**A**) and non-reduced (**B**) conditions. Both gels were stained with Coomassie Blue dye. A western blot under reduced (**C**) and non-reduced (**D**) conditions was performed with serum derived from one patient infected with *P. vivax*. The membranes were revealed after incubation with goat anti-human total IgG conjugated to HRP. The hybrid mAbs or recombinant proteins are presented in the following order: (1) αDEC, (2) αDEC-MSP1_19_, (3) αDEC-MSP1_42_, (4) recombinant MSP1_19_ protein, and (5) recombinant MSP1_33_ protein. Note that the recombinant proteins were not included in the non-reducing gels. The numbers displayed on the left of each gel indicate the molecular weights in kDa that were cropped out of the final figure. (**E**) Five million splenocytes from C57BL/6 mice were incubated on ice with 10, 1 or 0.1 μg/mL of the hybrid αDEC-MSP1_42_, αDEC-MSP1_19_ or αDEC mAbs. Splenocytes were then incubated with a pool of fluorescent antibodies and gated as singlets and CD19^−^DX5^−^. DCs were selected as CD11c^+^MHCII^+^, and subsequently divided in CD8α^+^ (DEC205 expressing subset) and CD8α^−^. Binding was detected on 1 × 10^6^ cells using an anti-mouse IgG1-PE antibody. One experiment representative of three is shown. Analysis was performed using FlowJo software.

For the production of αDEC-MSP1_42_ and αDEC-MSP1_19_ mAbs, the open reading frames from *msp1*_*19*_ or *msp1*_*42*_ genes were cloned in frame with the carboxyl-terminal portion of the heavy chain of the αDEC205 mAb exactly as described in methods and in ref. 14. As a control, we also produced the αDEC205 mAb without any fused antigen. Of note, all our attempts to produce an isotype control fused with the MSP1_42_ protein failed, as the hybrid mAb came out very degraded (data not shown). After transient transfection, the αDEC hybrid mAbs were purified and their integrity was evaluated by SDS-PAGE under reducing and non-reducing conditions. The recombinant proteins MSP1_19_ and MSP1_33_ were also produced and evaluated in the same manner (Fig. 1). A reduced gel showed that the heavy chains of the hybrid mAbs had the expected electrophoretic motilities (~92 kDa for αDEC-MSP1_42_, ~69 kDa for αDEC-MSP1_19_ and ~50 kDa for αDEC, Fig. 1A). All light chains migrated at ~25 kDa. Recombinant (rec.) MSP1_19_ and MSP1_33_ proteins migrated at ~19 kDa and ~33 kDa, respectively. The non-reduced gel showed mainly the presence of a single band for each of the mAbs (Fig. 1B).

In an attempt to verify if both hybrid mAbs and recombinant proteins retained antigenicity, a western blot was performed using sera from a *P. vivax* infected patient. Figure 1 shows that the patient’s serum recognized the heavy chains (Fig. 1C), or the entire hybrid mAbs (Fig. 1D) containing MSP1_42_ or MSP1_19_. Recognition was also observed for the rec. MSP1_19_ and rec. MSP1_33_ proteins (Fig. 1C).

To verify if the addition of either MSP1_42_ or MSP1_19_ to the αDEC205 mAb altered its binding to the DEC205 receptor, different concentrations (10, 1 and 0.1 μg/mL) of the αDEC-MSP1_42_ or αDEC-MSP1_19_ mAbs were incubated with splenic CD11c^+^CD8α^+^ (DCs that express the DEC205 receptor^17^) or with CD11c^+^CD8α^−^ DCs (Fig. 1E), or with CHO cells expressing either the murine or the human DEC205 receptors (Supplementary Fig. 1). The empty αDEC205 mAb was used as control. All mAbs bound specifically and in a dose dependent manner to the CD11c^+^CD8α^+^ DCs in the spleen (Fig. 1E, CD11c^+^CD8α^+^ panel) or to CHO cells expressing the murine DEC205 receptor (Supplementary Fig. 1A). No binding was observed to the CD11c^+^CD8α^−^ DCs that do not express the DEC205 receptor (Fig. 1E, CD11c^+^CD8α^−^ panel) or to CHO cells expressing the human DEC205 (Supplementary Fig. 1B).

### Targeting of the MSP1_42_ protein to the DEC205^+^ DC population increases the anti-MSP1_19_ antibody response, is dependent on CD4^+^ T cell help and promotes class switch and affinity maturation

**Figure 2:**
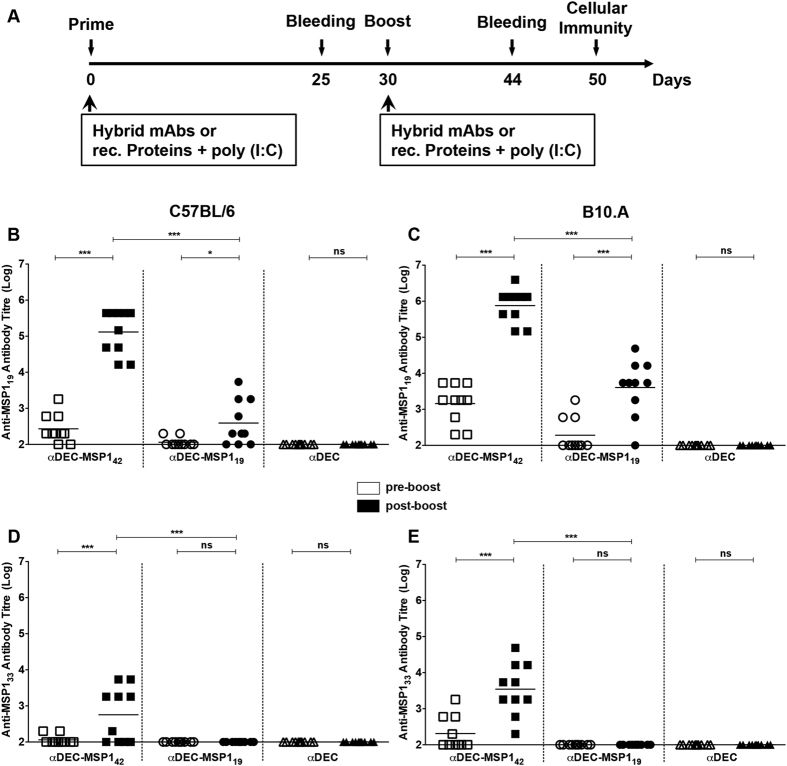
Immunization with hybrid αDEC-MSP1_42_ mAb induces higher anti-MSP1_19_ antibody titres when compared to immunization with αDEC-MSP1_19_ mAb. (**A**) Groups of C57BL/6 or B10.A mice (n = 10) were immunized with 5 μg of hybrid αDEC-MSP1_42_, αDEC-MSP1_19_ or αDEC in the presence of 50 μg of poly (I:C). Thirty days after the first dose, the animals received a booster dose in the same conditions as priming. The anti-MSP1_19_ or anti-MSP1_33_ IgG responses were measured by ELISA 5 days before (pre-boost) and 14 days after administration of the booster dose (post-boost). Total anti-MSP1_19_ IgG antibodies were detected in C57BL/6 (**B**) and B10.A (**C**) mice. Anti-MSP1_33_ antibody detection was also performed in C57BL/6 (**D**) and B10.A (**E**) mice. Graphs show the antibody titres of different groups normalized in log10 scale. Animals are represented individually (n = 10/group). Experiments were analysed by one-way ANOVA followed by the post-test HSD Tukey. P-value indicators * and *** refer to p < 0.05 and p < 0.001, respectively, while ns = not significant.

**Figure 3:**
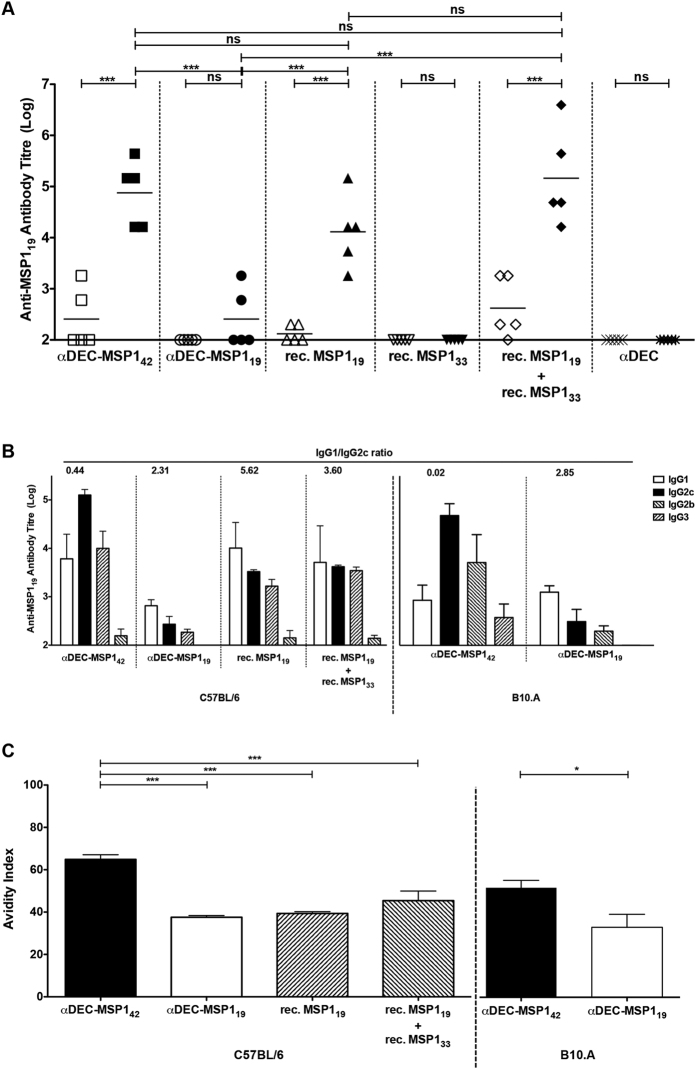
Immunization with αDEC-MSP1_42_ promotes class switch and affinity maturation. (**A**) Groups (n = 5) of C57BL/6 mice were immunized as described in Fig. 2 with αDEC-MSP1_42_, αDEC-MSP1_19_, αDEC, rec. MSP1_19_, rec. MSP1_33_ or with a combination of rec. MSP1_33_+MSP1_19_ in the presence of poly (I:C). The anti-MSP1_19_ antibody titres were measured by ELISA before and after the administration of the booster dose. Graph shows the antibody titres of different groups normalized in log10 scale, and animals are represented individually. (**B**) The anti-MSP1_19_ antibody titres for each IgG subclass (IgG1, IgG2b, IgG2c and IgG3) were determined 14 days after the administration of the booster dose in groups of C57BL/6 or B10.A mice. Graphs show the antibody titres plotted in log10 scale. Each bar represents the mean values ± SD of the antibody titres obtained for 5 mice. Numbers above the bars indicate the IgG1/IgG2c ratio calculated for αDEC-MSP1_42_ and αDEC-MSP1_19_ immunized groups. Results are representative of 2 independent experiments. (**C**) ELISA assessed anti-MSP1_19_ antibody avidities using 7 M urea for 5 min. The avidity index was calculated as the ratio between the OD_490_ obtained after and before urea treatment multiplied by 100. Results are expressed by the mean ± SD of two distinct experiments performed in triplicates. One-way ANOVA followed by the post-test HSD Tukey was performed. *** Refers to p < 0.001 and ns = not significant. Results are representative of 2 independent experiments.

In an attempt to study the anti-MSP1_19_ antibody response elicited when both hybrid mAbs were administered to mice, two different strains were used throughout this study: C57BL/6 (H-2b haplotype) and B10.A (H-2a(k/d) haplotype). Groups of C57BL/6 or B10.A mice received two doses containing 5 μg of each hybrid mAb or the empty αDEC administered in a 30-day interval in the presence of poly (I:C). Figure 2A shows a schematic representation of the immunization protocol. Five days before or 14 days after the administration of the second dose, mice were bled and anti-MSP1_19_ or anti-MSP1_33_ antibody titres were measured by ELISA (Fig. 2B–E). In both mouse strains, the antibody titres against MSP1_19_ increased after the administration of the second dose (Fig. 2B and C) in mice immunized either with αDEC-MSP1_42_ or αDEC-MSP1_19_. No anti-MSP1_19_ titres were detected in the group immunized with αDEC. Of note was the fact that in both mouse strains, the amount of anti-MSP1_19_ antibodies induced in mice that received αDEC-MSP1_42_ mAb was approximately 100x higher than in mice that received αDEC-MSP1_19_ mAb. As expected, anti-MSP1_33_ antibodies were only detected in mice immunized with αDEC-MSP1_42_ (Fig. 2D and E). To compare the anti-MSP1_19_ antibody response in the presence or absence of DEC205^+^ DC targeting, we immunized groups of C57BL/6 mice with αDEC-MSP1_42_, αDEC-MSP1_19_, αDEC, rec. MSP1_19_, rec. MSP1_33_ or with a combination of rec. MSP1_33_ and MSP1_19_ (Fig. 3). To our surprise, mice immunized with rec. MSP1_19_ were able to produce anti-MSP1_19_ antibodies that, despite slightly lower, were not statistically different from those observed in mice immunized with αDEC-MSP1_42_ (Fig. 3A). No difference was also observed between the αDEC-MSP1_42_ and rec. MSP1_19_+MSP1_33_ groups. To better characterize the anti-MSP1_19_ response, the IgG subtypes were analysed in both mouse strains and the IgG1/IgG2c ratio was calculated (Fig. 3B). Both C57BL/6 and B10.A mice presented detectable levels of all IgGs in the group immunized with αDEC-MSP1_42_ mAb. An analysis of the IgG1/IgG2c ratio showed that the response in both mouse strains was prone to Th1 (IgG1/IgG2c < 1). On the other hand, in the group immunized with αDEC-MSP1_19_ (in both mouse strains) or with rec. MSP1_19_ or rec. MSP1_19_+MSP1_33_ (in C57BL/6 mice), a more Th2 type of response was observed (IgG1/IgG2c > 1).

The detection of all IgG subclasses in the anti-MSP1_19_ antibody response in both mouse strains, and especially in the αDEC-MSP1_42_ immunized group, suggested that B cells were undergoing class switching and probably affinity maturation. To test this hypothesis, we measured the avidity index of the anti-MSP1_19_ antibodies in C57BL/6 and B10.A mice immunized with the hybrid mAbs or with the rec. proteins (Fig. 3C). We observed that anti-MSP1_19_ antibodies induced in both mouse strains immunized with αDEC-MSP1_42_ showed a higher avidity index when compared to the group immunized with αDEC-MSP1_19_ (Fig. 3C). More importantly, the avidity index presented by C57BL/6 mice immunized with αDEC-MSP1_42_ was higher than the observed in groups that received rec. MSP1_19_ or rec. MSP1_33_+MSP1_19_ proteins (Fig. 3C). Taken together, these results suggest that MSP1_42_ targeting to the DEC205^+^ DC subset is able to alter the quality of the anti-MSP1_19_ humoral immune response.

**Figure 4:**
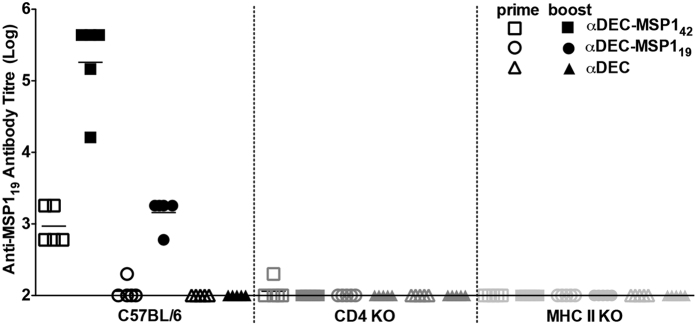
The anti-MSP1_19_ antibody response induced by the immunization with hybrid mAbs is dependent on CD4^+^ T cell help. Groups (n = 5) of C57BL/6, CD4 KO and MHCII KO mice were immunized as described in Fig. 2. The anti-MSP1_19_ antibody titres were measured by ELISA before and after the administration of the booster dose. Graph shows the antibody titres of different groups normalized in log10 scale, and animals are represented individually. Results are representative of 2 independent experiments.

It was previously shown that antigen targeting to the DEC205^+^ DCs induces an antibody response that requires T cell help^5^. To confirm this requirement in our model, we immunized WT, CD4 KO and MHCII KO mice (C57BL/6 background) with our hybrid mAbs (Fig. 4). As expected, the anti-MSP1_19_ response was abolished in the absence of CD4^+^ T cells or MHCII presentation.

To test if the anti-MSP1_19_ antibodies could bind to the MSP1_19_ protein on the surface of cells, we transfected HEK293T cells with a plasmid capable of expressing the MSP1_19_ as a transmembrane protein (Supplementary Fig. 2A). As a negative control, HEK293T cells were also transfected with a plasmid containing the unrelated Duffy binding protein II (DBPII, Supplementary Fig. 2B). Besides MSP1_19_ or DBPII proteins, both plasmids also expressed the green fluorescence protein (GFP). GFP^+^ cells were gated for the analysis (Supplementary Fig. 2C and D). We observed that anti-MSP1_19_ antibodies induced in mice immunized with either αDEC-MSP1_42_ or αDEC-MSP1_19_ in both mouse strains (Supplementary Fig. 2E for C57BL/6, and 2G for B10.A) bound to the MSP1_19_ expressing cells while no significant ligation was observed in the DBPII transfected cells (Supplementary Fig. 2F for C57BL/6, and 2H for B10.A). It is important to mention that to perform this assay we normalized the amount of anti-MSP1_19_ antibodies present in the sera.

In summary, the results presented above show that immunization with αDEC-MSP1_42_ is able to induce stronger and broader humoral immune response when compared with αDEC-MSP1_19_, in two different mouse strains.

### MSP1_42_ targeting to DEC205^+^ DCs induces activation and proliferation of CD4^+^ T cells specific for the 33-kDa fragment

**Figure 5:**
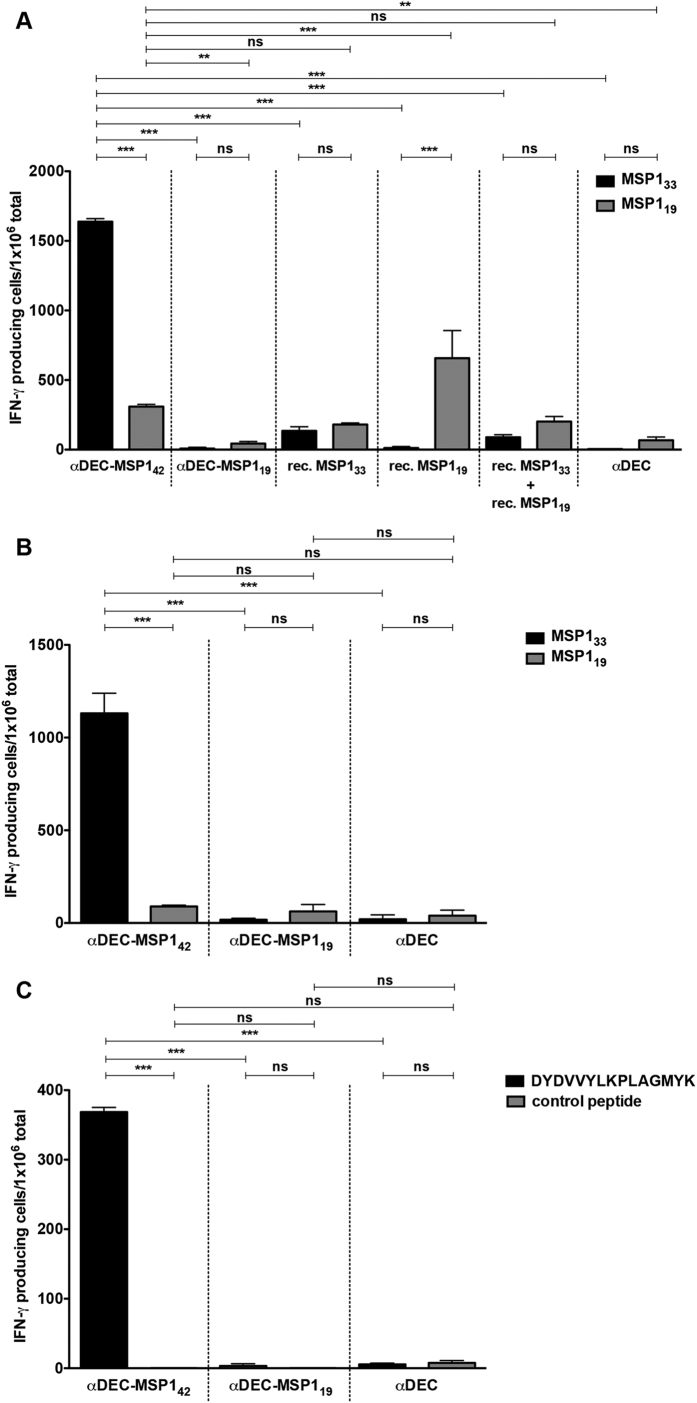
Splenocytes from αDEC-MSP1_42_ immunized mice produce IFN-γ in response to MSP1_33_ recombinant protein. Groups of C57BL/6 (**A**) and B10.A (**B** and **C**) mice (n = 3) were immunized as described in Fig. 2. The cellular immune response was evaluated by ELISPOT 20 days after administration of the booster dose. Total splenocytes were stimulated with 1 μg/ml of recombinant MSP1_33_ or MSP1_19_ proteins (**A** and **B**) or with the DYDVVYLKPLAGMYK or a control unrelated peptide (**C**). Graphs show the number of IFN-γ producing cells per million cells after subtracting the number of IFN-γ producing cells obtained in the absence of any stimulus. The experiment was performed in triplicates using samples from pooled mice. Bars indicate mean ± SD and the experiment was analysed by one-way ANOVA followed by the post-test HSD Tukey. P-value indicators ** and *** refer to p < 0.01 and p < 0.001, respectively, while ns = not significant. Results are representative of 3 independent experiments.

The results described above suggested that MSP1_42_ sequence carried epitopes that might provide help for the B cell mediated antibody production when targeted to the DEC205^+^ DCs. To start mapping the important MSP1_42_ regions, we performed ELISPOT assays to detect IFN-γ producing cells using splenocytes from C57BL/6 mice immunized with αDEC-MSP1_42_, αDEC-MSP1_19_, αDEC, rec. MSP1_19_, rec. MSP1_33_ or with rec. MSP1_33_+MSP1_19_ (Fig. 5A), or from B10.A immunized with αDEC-MSP1_42_, αDEC-MSP1_19_ or αDEC (Fig. 5B). We detected large numbers of IFN-γ producing cells only in splenocytes derived from mice immunized with αDEC-MSP1_42_ mAb and pulsed with recombinant MSP1_33_. This was observed in C57BL/6 (Fig. 5A) and in B10.A (Fig. 5B) mice. Pulse with rec. MSP1_19_ induced fewer IFN-γ producing cells in C57BL/6 and in B10.A, indicating that the immunodominant T cell epitopes are probably present in the 33-kDa portion of MSP1_42_. Of note, we were able to detect a reasonable number of IFN-γ producing cells in C57BL/6 mice immunized with the rec. MSP1_19_ but not in the animals immunized with αDEC-MSP1_19_ when the splenocytes were pulsed with the same protein. To further analyse the response, we took advantage of the fact that T cells from B10.A mice recognize peptide DYDVVYLKPLAGMYK previously described^37^. We then pulsed splenocytes from αDEC-MSP1_42_, αDEC-MSP1_19_ or αDEC immunized mice with the DYDVVYLKPLAGMYK peptide and observed that only cells derived from αDEC-MSP1_42_ mice were able to produce IFN-γ (Fig. 5C). However, the number detected (~380 per 10^6^ total splenocytes) was smaller than the number obtained when the pulse was performed with the recombinant MSP1_33_ protein (~1,100 per 10^6^ total splenocytes), indicating that there are probably other epitopes that still need to be mapped.

**Figure 6:**
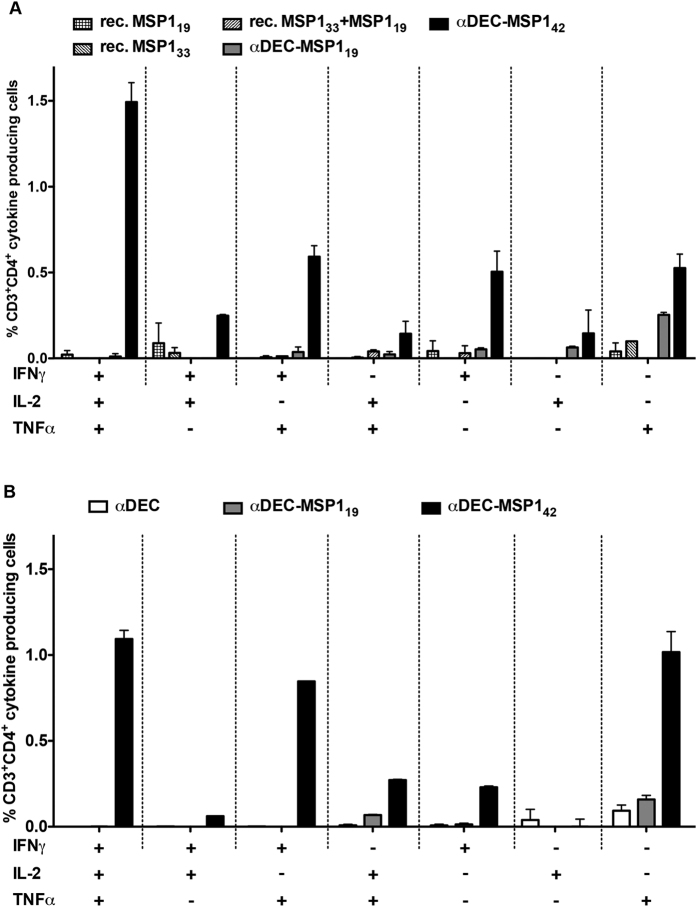
Immunization with the αDEC-MSP1_42_ mAb induces the polyfunctional CD4^+^ T cells after restimulation with MSP1_33_ recombinant protein in C57BL/6 and B10.A mice. Groups of mice (n = 3) were immunized as described in Fig. 2. IFN-γ, IL-2 and TNFα were detected by intracellular staining 20 days after the administration of the booster dose in C57BL/6 (**A**) and B10.A (**B**) mice. Splenocytes were stimulated with 5 μg/ml of MSP1_33_ recombinant protein. Graphs show the percentage of cells producing IFN-γ, IL-2 and/or TNFα in the CD3^+^CD4^+^ gate after subtracting the values obtained in the absence of any stimulus. Boolean combinations were created using FlowJo software to determine the frequency of each cytokine production based on all possible combinations of cytokine expression. The experiment was performed in duplicates using samples from pooled mice. Results are representative of 2 independent experiments.

**Figure 7:**
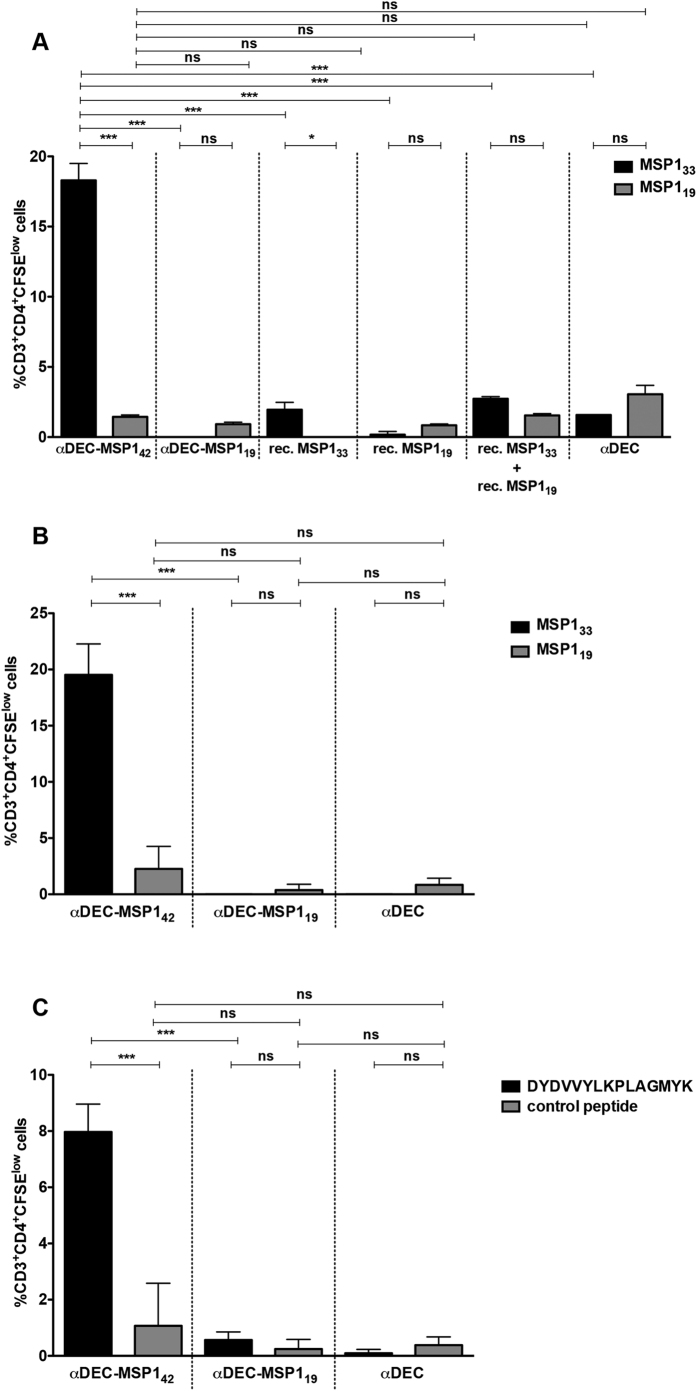
MSP1_42_ targeting to the DEC205^+^ DC population stimulates CD4^+^ T cell proliferation in response to MSP1_33_ protein. Groups of mice (n = 3) were immunized as described in Fig. 2. Twenty days after the administration of the second dose, total splenocytes from C57BL/6 (**A**) and B10.A (**B** and **C**) mice were labelled with CFSE and placed in culture in the presence or absence of 5 μg/ml of MSP1_33_ or MSP1_19_ recombinant proteins. Graphs show the percentage of CD3^+^CD4^+^ T cells that lost CFSE (CFSE low) after subtracting the values obtained in the absence of any stimulus. The experiment was performed in triplicates using samples from pooled mice. Bars indicate mean ± SD and the experiment was analysed by one-way ANOVA followed by the post-test HSD Tukey. *** Refers to p < 0.001, ns = not significant. Results are representative of 2 independent experiments.

To further investigate the cellular immune response induced in mice immunized with αDEC-MSP1_42_ mAb, we evaluated the production of three inflammatory cytokines: IFN-γ, IL-2 and TNFα by CD4^+^ (Supplementary Fig. 3) and CD8^+^ T cells (data not shown). CD4^+^ T cells derived from either C57BL/6 or B10.A mice immunized with αDEC-MSP1_42_ were able to produce the three cytokines when restimulated only with MSP1_33_ recombinant protein. Restimulation with recombinant MSP1_19_ was unable to elicit significant percentages of CD4^+^ T cells producing IFN-γ, IL-2 or TNFα in C57BL/6 (Supplementary Fig. 3A) or B10.A mice (Supplementary Fig. 3B). Interestingly, very low levels of CD4^+^ T cells producing any cytokine were observed in C57BL/6 immunized with rec. MSP1_19_ or rec. MSP1_33_ (Supplementary Fig. 3A). Although surprising, we did not detect specific responses elicited in CD8^+^ T cells (data not shown). Using boolean gating analysis, we were able to detect the simultaneous production of the inflammatory cytokines IFN-γ, IL-2 and TNFα by CD4^+^ T cells (Fig. 6). We observed that CD4^+^ T cells derived from mice immunized with αDEC-MSP1_42_ mAb were able to produce combinations of the three tested cytokines in both mouse strains (Fig. 6A, C57BL/6 and 6B, B10.A) when pulsed with MSP1_33_ recombinant protein. In fact, in both mouse strains we were able to detect polyfunctional CD4^+^ T cells producing all combinations of the three cytokines tested (Fig. 6), especially those producing all three at the same time. To access T cell proliferation, splenocytes from mice immunized with the hybrid mAbs or rec. proteins were stained with CFSE and pulsed with either recombinant MSP1_19_ or MSP1_33_ proteins (Fig. 7). CD4^+^ T cell proliferation was observed in both mouse strains (Fig. 7A, C57BL/6 and 7B, B10.A) mainly in the animals immunized with αDEC-MSP1_42_ pulsed with recombinant MSP1_33_. CD8^+^ T cell proliferation was not observed in any case (data not shown). In addition, we observed that CD4^+^ T cells from B10.A mice also proliferated in response to the DYDVVYLKPLAGMYK peptide (Fig. 7C). However, the percentage of CD3^+^CD4^+^ T cells that responded to this peptide was smaller than that obtained when recombinant MSP1_33_ was used (Fig. 7B) indicating the presence of other T cell epitopes in the MSP1_33_ protein sequence.

The results presented above show that antigen targeting to the DEC205^+^ DC population in the presence of poly (I:C) is effective in inducing potent antibody and CD4^+^ T cell responses when epitopes are present in the protein structure. In the absence of such epitopes, the response is weak.

## Discussion

Antigen targeting to the CD8α^+^ DC population through the use of αDEC205 hybrid mAbs has been successfully used in different models, and was shown to induce both CD8^+^ and CD4^+^ T cells^5^,^6^,^7^,^13^,^15^,^17^,^18^,^19^,^20^,^21^,^22^,^23^,^24^. In addition, the induction of antibodies against the fused antigen was also reported previously^3^,^5^. All these studies used antigens previously described as immunogenic. However, to our knowledge, no one has yet targeted different fragments of the same antigen to the DEC205^+^ DCs, and asked what would be the immune response outcome. To start addressing that question, we produced two mAbs containing fragments of the MSP1 protein derived from *P. vivax*. The αDEC-MSP1_19_ mAb contains the C-terminal 19-kDa fragment, which is normally target of antibodies in infected individuals^28^,^36^,^38^, while the αDEC-MSP1_42_ mAb contains the 19-kDa fragment (MSP1_19_) fused with the 33-kDa (MSP1_33_) fragment that was shown to elicit T cell responses in the field^31^. Of note, it was not evaluated which epitopes were recognized by CD4^+^ or CD8^+^ T cells^31^. Both hybrid αDEC-derived mAbs were produced successfully, retained antigenicity and were able to target the DEC205 receptor expressed on the surface of CD8α^+^ DC population. This is not the first time a *Plasmodium* derived antigen is fused to the αDEC205 mAb. Previously, the circumsporozoite protein (CSP) from *P. yoelii* and *P. falciparum* was also used to immunized mice^5^ and non-human primates^24^. However, to our knowledge, this is the first time an antigen expressed by the erythrocytic stage (merozoites) is fused to the αDEC205 mAb. We took advantage of the fact that MSP1_42_ contains MSP1_19_, and immunized mice with both αDEC-derived hybrid mAbs in an attempt to study the anti-MSP1_19_ antibody response elicited when MSP1_19_ was targeted alone or fused to MSP1_33_. As mentioned previously, we were unable to produce an isotype control fused to the MSP1_42_ protein, and then used the recombinant proteins as non-targeted controls. The use of poly (I:C) as a DC maturation stimulus is well documented in the literature and it seems to be very potent when administered together with αDEC205 fusion mAbs^3^,^7^,^14^,^15^,^24^,^39^. An increase in either anti-MSP1_19_ or anti-MSP1_33_ antibody titres was observed after the administration of the second dose, which has been consistently observed in other models^5^,^14^. Interestingly, the anti-MSP1_19_ antibody titres were increased about 100x when MSP1_42_ was targeted to the CD8α^+^ DC population in two different mouse strains. This result suggested that the presence of MSP1_33_ fragment was helping to increase the anti-MSP1_19_ antibody response possibly because of the presence of additional T cell epitopes in the region of 33 kDa. However, to our surprise, immunization with rec. MSP1_19_ or with rec. MSP1_33_+MSP1_19_ was able to induce high anti-MSP1_19_ antibody titres in C57BL/6 mice that were not different from those induced by αDEC-MSP1_42_. This result was unexpected but may reflect a longer persistence of rec. MSP1_19_ in the circulation leading to an increase in the antigen uptake by B cells. We also cannot rule out the possibility that MSP1_19_ contains minor CD4^+^ T cell epitopes responsible for helping B cells to produce antibodies when the protein is not directly targeted to the DEC205^+^ DCs. The dependency of T cell help for the induction of antibody responses after targeting to DEC205^+^ DCs was shown after immunization of CD4 and MHCII KO mice, when complete abrogation of the antibody response was observed in the animals. To characterize in more detail the anti-MSP1_19_ antibody response, we carefully analysed the IgG subclasses induced when the hybrid mAbs or the rec. proteins were used. Surprisingly, immunization with the αDEC-MSP1_42_ mAb induced a very different profile of IgG subclasses when compared to either αDEC-MSP1_19_ or rec. proteins immunized mice. The IgG1/IgG2c ratio in animals immunized with αDEC-MSP1_42_ was <1, indicating a more prone Th1-type of response in both mouse strains analysed. On the other hand, the group immunized with αDEC-MSP1_19_ or with the rec. proteins showed a higher IgG1/IgG2c ratio (>1) in both mouse strains. Previous reports have shown that antigen targeting through DEC205 is able to elicit high titres of IgG2c (or IgG2a, depending on the mouse strain)^5^,^14^,^23^. These results also suggested that immunization with αDEC-MSP1_42_ could be inducing a more pronounced class switch that would imply in an increase in affinity maturation. We then measured the avidity index of the polyclonal sera induced in mice immunized with αDEC-MSP1_42_, αDEC-MSP1_19_ or with the rec. proteins. In both mouse strains, the avidity index was higher in the sera of animals immunized with the αDEC-MSP1_42_ mAb, suggesting that the anti-MSP1_19_ specific B cells were undergoing affinity maturation particularly in this group of mice. On the other hand, the ability to bind to MSP1_19_ expressed on the surface of transiently transfected HEK293T cells was similar between the groups immunized with either αDEC-MSP1_42_ or αDEC-MSP1_19_ mAbs, when we considered dilutions where both sera presented similar OD values. Despite the similar binding observed when similar amounts of anti-MSP1_19_ antibodies were used, the fact that immunization with αDEC-MSP1_42_ induces higher antibody titres with increased avidity may be favourable in the field where higher anti-MSP1_19_ titres have been associated with protection against malaria^40^.

The results involving the anti-MSP1_19_ antibody response described above indicated that CD4^+^ T cells were being activated during immunization with the αDEC-MSP1_42_ mAb, as the initial T-B cell interaction leads to germinal centre formation, affinity maturation, and isotype switching. The increased production of IgG2c prompted us to investigate if T cells were able to produce IFN-γ, a cytokine associated with the production of this subclass^41^. We were able to detect high numbers of IFN-γ producing T cells in both mouse strains immunized with αDEC-MSP1_42_ mAb only when splenocytes were pulsed with the recombinant MSP1_33_ protein. This indicated that the immunodominant epitopes were restricted to the 33-kDa fragment of the molecule. Interestingly, we did not observe a noticeable response when the rec. MSP1_33_ was used as immunogen. On the other hand, we were able to detect IFN-γ producing T cells in animals immunized with rec. MSP1_19_ whose splenocytes were pulsed with the same protein. In addition, we found that CD4^+^ T cells induced by immunization with αDEC-MSP1_42_ mAb were able to produce different combinations of three inflammatory cytokines (IFN-γ, IL-2 and TNFα), and proliferate, when pulsed with the MSP1_33_ recombinant protein. These results showed that the presence of immunodominant epitopes in the 33-kDa fragment of MSP1 activate polyfunctional CD4^+^ T cells and the anti-MSP1_19_ antibody response. In B10.A mice, we were able to detect a specific response against the previously defined DYDVVYLKPLAGMYK epitope^37^, but more epitopes are probably present on the MSP1_33_ sequence as this response was weaker than that observed with the recombinant MSP1_33_ pulse. To completely map the immunodominant epitopes, additional experiments using peptide libraries will be necessary. Induction of CD4^+^ T cell proliferation and cytokine production after immunization with a hybrid αDEC205 mAb in the presence of poly (I:C) has been reported previously by Trumpfheller *et al*.^15^. The use of a αDEC205 mAb fused to a HIV protein elicited mainly CD4^+^ T cells that proliferated and produced the three inflammatory cytokines (IFN-γ, IL-2 and TNFα). In our case, we detected CD4^+^ T cells that proliferated vigorously after recombinant MSP1_33_ pulse and were capable of producing not only three cytokines simultaneously, but also two or one. On the other hand, immunization with αDEC-MSP1_19_ mAb or with the rec. proteins was extremely inefficient in inducing either proliferation or cytokine production. These results highlight the importance of the antigen choice when targeting the DEC205 receptor on the surface of CD8α^+^ DCs.

Of note, we have to mention that contrary to other results previously reported on the literature^5^,^6^,^7^,^13^,^21^, we did not detect CD8^+^ T cell proliferation or cytokine production when MSP1_42_ or MSP1_19_ were targeted to the DEC205^+^ DCs. The simpler explanation may be that *P. vivax* MSP1_42_ sequence does not contain CD8^+^ T cell epitopes or those epitopes are not recognized by C57BL/6 and B10.A haplotypes. We were unable to find in the literature any previous reports mapping CD8^+^ T cell epitopes to both *P. vivax* MSP1_42_ and MSP1_19_ proteins. However, we found one report that described CD8^+^ T cell epitopes in the sequence of the murine *P. yoelii* MSP1_42_^33^. It is important to mention that the amino acid sequences of MSP1 from *P. vivax* and *P. yoelii* are quite distinct, and that the CD8^+^ T cells were detected in BALB/c mice after immunization with a recombinant adenovirus. Besides, CD8^+^ T cells were detected after splenocytes were pulsed with peptide pools^33^. Indeed, almost every time CD8^+^ T cells were detected after antigen targeting to CD8α^+^DEC205^+^ DCs, detection was measured using overlapping peptide libraries or previously described peptides, and frequently previously enriched CD8^+^ T cells^5^,^6^,^7^,^21^. In this work, we did not use overlapping peptide libraries or CD8^+^ T cell enrichment. Instead, bulk splenocytes were pulsed directly with the rec. proteins. Another explanation that may account for the absence of CD8^+^ T cell detection may have to do with the timing of analysis. Here we analysed cellular immune responses on day 20 after boost while others normally analyse T cell responses at earlier time points^16^. In an attempt to maximize our window of detection, we analysed the animals on day 5 after the boost, and still could not detect CD8^+^ T cell proliferation or cytokine production (data not shown). However, we still cannot rule out the possibility of CD8^+^ T cell activation because we did not enrich the T cells or used peptide libraries. In fact, we plan to explore those possibilities in the future. Finally, it is important to point out that MSP1_42_ is an antigen expressed during the erythrocytic phase of *Plasmodium* life cycle, and the evidence points to a role of antibodies and CD4^+^ T cells in protection, while CD8^+^ T cells would have a more pronounced role during the pre-erythrocytic phase, before the parasites reach the blood.

Taken together, our results show that the choice of the antigen may be important when designing vaccines targeted to the CD8α^+^DEC205^+^ DC subset.

## Material and Methods

### Mice

Six- to 8-week-old female C57BL/6 and C57BL/6 CD4 KO mice were bred at the Isogenic Mouse Facility of the Parasitology Department, University of São Paulo, Brazil. Female B10.A mice were obtained from the Isogenic Mouse Facility of the Immunology Department, University of São Paulo, Brazil. Female C57BL/6 MHCII KO mice were obtained from the Division of Immunology, Federal University of São Paulo (UNIFESP). All protocols were approved by the Institutional Animal Care and Use Committee (CEUA) of the University of São Paulo (protocol number 082) and all the animals were handled according to the Brazilian College of Animal Experimentation guidelines. In addition, all experimental methods were performed in accordance with the National Institutes of Health Guide for the Care and Use of Laboratory Animals and the Brazilian National Law (11.794/2008).

### Plasmid generation

The sequence encoding aminoacids 1326 to 1705 from the *P. vivax* MSP1 protein (Belem strain, accession number AF435594.1^42^) was synthesized by GenScript (Piscataway, NJ, USA) with codon optimization for expression in mammalian cells. This sequence corresponds to the 42 kDa portion of the MSP1 protein (MSP1_42_). The three putative glycosylation sites (NIT, NES and NVT) were substituted by NII, EES and DVT. Amplification was accomplished using the Phusion High Fidelity DNA Polymerase (New England Biolabs) according to the manufacturer’s instructions. A 1,140 bp fragment was amplified, cloned into the pJET 1.2/blunt vector (ThermoScientific) and then digested with the restriction enzymes *Xho* I and *Not* I (New England Biolabs). After digestion, the fragment was ligated in frame with the carboxyl terminus of the heavy chain of mouse αDEC205 (NLDC145 clone) or with an isotype control (GL117 clone) mAb (kindly provided by Dr. Michel C. Nussenzweig, The Rockefeller University), as previously described^14^. In addition, a 267 bp fragment (amino acids 1617 to 1705) corresponding to the 19 kDa portion of the MSP1 protein (MSP1_19_) was also amplified and cloned as described above. The final plasmids were named pDEC-MSP1_42_, Iso-MSP1_42_, pDEC-MSP1_19_ and Iso-MSP1_19_, and sequenced to confirm the presence of either MSP1_42_ or MSP1_19_ sequences in frame.

For the production of the recombinant MSP1_19_ protein, we used the plasmid pET14b-MSP1_19_ previously described by ref. 43, while the sequence corresponding to amino acids 1326 to 1616 was amplified by PCR as described above and cloned into the pET28a vector. Plasmid pET28a-MSP1_33_ was then generated.

#### Expression of recombinant hybrid mAbs and proteins

Plasmids containing the heavy chain of the mouse αDEC205 mAbs (pDEC-MSP1_19_, pDEC-MSP1_42_, or pDEC without any fused antigen) or the isotype controls and the respective light chains (pDEC kappa, kindly provided by Dr. Michel C. Nussenzweig, The Rockefeller University, New York, USA) were amplified in DH5α bacteria, and subsequently purified in large scale using the QIAGEN Maxi Prep kit (Qiagen), according to the manufacturer’s instructions.

Transient transfection in human embryonic kidney (HEK) 293T (ATCC No CRL-11268) cells was performed exactly as described in ref. 14. The recombinant fusion mAbs were purified with the aid of Protein G beads (GE Healthcare) according to the manufacturer’s instructions. After purification, all the fractions containing antibodies were pooled together, dialysed against 2 L cold PBS, and sterilized filtered through 0.2 μm membranes (TPP). The fusion mAbs αDEC-MSP1_42_, αDEC-MSP1_19_ and αDEC had their concentrations estimated by Bradford assay (Pierce), and an assay for the detection of LPS (QCL -1000, Lonza) was performed after purification of each batch. Samples containing less than 1 EU/mL were considered clean, and aliquots were stored at −20 °C until use. We were unable to produce the fusion mAb Iso-MSP1_42_ as all our attempts resulted in degradation.

The recombinant MSP1_19_ protein was produced according to the protocol described in ref. 43. A different protocol was developed for the production of the recombinant MSP1_33_ protein. Briefly, a 125 ml bacterial pellet was dissolved in 5 ml of lysis buffer (50 mM Tris, 200 mM Nacl, 10% glycerol, pH 8.0). Next, PMSF to a final concentration of 1 mM was added and the solution was centrifuged at 28,000 × g for 45 minutes. The supernatant was incubated with 5 ml of Ni-NTA (Quiagen) previously equilibrated in lysis buffer for 2 hours at 4 °C, under rotation. After 4 washes with 10 ml of wash buffer (50 mM Tris, 500 mM NaCl, 10% glycerol, 30 mM imidazole, pH 8.0), the protein was eluted from the Ni-NTA matrix using 5 ml of elution buffer (50 mM Tris, 1 M Nacl, 10% glycerol, 500 mM imidazole pH 8.0) for 1 hour at 4 °C.

#### Immunoblots

Approximately 1 μg of each fusion mAb or the recombinant protein were resolved on 7 or 12% SDS-PAGE gels under non-reducing or reducing conditions, respectively. Gels were either stained with Coomassie Blue (Amresco) or transferred to nitrocellulose membranes (GE Healthcare). Coomassie Blue stained gels were then scanned using a Lexmark 3600–4600 series scanner and transformed into grayscale using AdobePhotoshop CC software (Adobe Systems Incorporated 2013). Nitrocellulose membranes were blocked for an 1 hour at room temperature (rt) in 0.05% PBS-Tween 20 (PBS-T), 5% non-fat milk and 1% BSA, and then incubated with serum (1:2,000 dilution) derived from a patient previously infected with *P. vivax* (kindly provided by Dr. Claudio R.F. Marinho, University of São Paulo, Brazil). After a 2-hour incubation at rt the membranes were washed twice and incubated for an additional hour using an anti-human IgG-HRP (1:5,000, Jackson Laboratories). After two additional washes, the membranes were developed using quimioluminescence (ECL kit, GE Healthcare) and captured on Kodak film. The films were then scanned and submitted to the same processing described above for Coomassie Blue gels.

#### Binding assay

Spleens from naïve mice were removed and splenocytes were obtained after erythrocyte lysis with ACK buffer (0.1 mM EDTA, 0.15 mM NH_4_Cl, 1 mM KHCO_3_). Five million splenocytes were incubated in PBS-FBS (fetal bovine serum) 2% containing Fc Block (anti-CD16/32, BD Biosciences) at a 1:100 dilution. After a 15-minute incubation on ice, αDEC-MSP1_42_, αDEC-MSP1_19_ or αDEC purified mAbs were diluted to 10, 1, 0.1 μg/ml and added to the wells. After 45 minutes of incubation, the cells were centrifuged and washed twice with PBS-FBS 2%. Another 45-minute incubation on ice followed in the presence of anti-mouse IgG1-PE (clone A85-1), anti-CD11c-APC (clone N418), anti-CD49b-biotin (Clone DX5), anti-CD19-biotin (clone1D3), anti-MHCII-FITC (2G9) and anti-CD8-PE-Cy7 (clone 53-6.7). After 2 more washes, cells were incubated on ice with Streptavidin-PerCP for 30 minutes. All mAbs were purchased from BD biosciences. Finally, after two final washes, half a million events were then read in a FACS Canto flow cytometer (BD biosciences), and analysed using FlowJo software (version 9.3, Tree Star, San Carlo, CA).

#### Immunization schedule

Groups of 5–10 female mice were immunized intraperitoneally with 5 μg of the following hybrid mAbs: αDEC-MSP1_42_, αDEC-MSP1_19_ or αDEC (as a negative control) in the presence of 50 μg of poly (I:C) (Invivogen). Groups of 5 female C57BL/6 also received 1.5 μg of rec. MSP1_33_ or 1 μg of rec. MSP1_19_ in the presence of the same amount of poly (I:C). This amount corresponds to the same number of molecules of either MSP1_33_ or MSP1_19_ present on 5 μg αDEC-MSP1_42_ mAb. Thirty days after the prime, animals received a second dose containing exactly the same amount of fusion mAbs and adjuvant. Animals were bled 5 days before or 14 days after the administration of the second dose and their sera were used for the analysis of the humoral response. Assays to evaluate the cellular immune response were performed on day 20 after the administration of the second dose, when the animals were euthanized.

#### Analysis of the antibody responses

For the detection of antibodies against either MSP1_19_ or MSP1_33_, sera from immunized mice were used in ELISA assays, exactly as described previously^14^. Briefly, high binding ELISA plates (Costar) were coated overnight at room temperature (rt) with 100 ng/well of MSP1_19_ or MSP1_33_ recombinant proteins diluted in PBS. After three washes and one-hour incubation in blocking buffer (PBS-Tween 20 0.02%, non-fat milk 5% and BSA 1%), sera were serially diluted in PBS-Tween 20 0.02%, non-fat milk 5% and BSA 0.25% and incubated for 2–3 h at rt. The secondary antibodies goat anti-mouse IgG Fc-specific-HRP (1:10,000; Jackson ImmunoResearch Laboratories) or anti-mouse IgG subclass-HRP specific antibodies (1:3,000; SouthernBiotech) were added after three additional washes. After one-hour incubation at rt, plates were vigorously washed and the enzymatic reaction was developed by the addition of 1 mg/ml of ortho-phenylenediamine dihydrochloride (Sigma) diluted in phosphate–citrate buffer, pH 5.0, containing 0.03% (v/v) hydrogen peroxide. Reactions were stopped using sulfuric acid 4N. OD_490_ was measured using a microplate reader (Biotek). Titres represent the highest serum dilution showing an OD_490_ ≥ 0.1 normalized in a log10 scale. The IgG1/IgG2c ratio was calculated by dividing the mean values of the highest serum dilution obtained for IgG1 by the mean value of the highest serum dilution obtained for IgG2c without normalization. The avidity index was calculated using an extra step of incubation with 7M urea for 5 min, exactly as described previously^44^,^45^.

### Analysis of T cell responses

#### Splenocyte isolation

After mice were euthanized, spleens were removed aseptically and processed exactly as described by ref. 14. Bulk splenocytes were ressuspended in R10 [RPMI supplemented with 10% of fetal bovine serum (GIBCO), 2 mM L-glutamine (GIBCO), 10 mM Hepes (GIBCO), 1 mM sodium pyruvate (GIBCO), 1% vol/vol non-essential aminoacid solution (GIBCO), 1% vol/vol vitamin solution (GIBCO), 20 μg/mL of ciprobacter (Isofarma, Brazil) and 5 × 10^−5^ M 2-mercaptoetanol (GIBCO)]. Cell viability was evaluated using 0, 1% Trypan Blue exclusion dye and cell concentration was estimated using a hemocytometer.

#### IFN-γ ELISPOT

ELISPOT assays for the detection of IFN-γ producing splenocytes were performed using the Ready-SET-Go kit (eBioscience), according to the manufacturer’s instructions. Three hundred thousand splenocytes were incubated in the presence of 1 μg/mL of the recombinant MSP1_19_ or MSP1_33_ proteins. Control cells were left unpulsed. The AEC kit (BD biosciences) was used to develop the spots that were counted with the aid of an automated stereomicroscope (KS ELISPOT, Zeiss, Oberkochem, Germany). The number of IFN-γ producing cells/10^6^ splenocytes was calculated after subtracting the number of cells in the unpulsed wells.

#### Detection of IFN-γ, IL-2 and TNFα producing cells by intracellular staining

Splenocytes isolated from immunized mice were obtained as described above, and plated in round-bottomed 96-well plates at a concentration of 1 × 10^6^ cells/well in triplicates. The cells were then incubated with the recombinant proteins MSP1_33_ or MSP1_19_ (5 μg/mL) in R10 medium containing 2 μg/mL of the αCD28 agonist antibody. As negative controls, some cells were left unpulsed while others were incubated with 1 μg/mL αCD3 as positive controls. After one-hour incubation at 37 °C and 5% CO_2_, 0.5 μg of Golgi Plug (Brefeldin A, BD Pharmingen) was added to each well and plates were re-incubated for another 12 hours. After this period, the plates were centrifuged for 5 min at 1,000 × g and the supernatant was discarded by inversion. Cells were then washed with PBS-FBS and transferred to V-bottomed 96-well plates. The cells were then surface stained with αCD4-PerCP-Cy5.5 mAb (clone RM 4–5) for 45 minutes on ice in PBS-FBS. After 3 washes, cells were resuspended in PharmingenStain buffer (BD Pharmingen) for 10 min on ice, centrifuged, and fixed and permeabilized using the Cytofix/Cytoperm kit (BD Pharmingen). After a 15-min incubation on ice, the plates were centrifuged and washed 3 times with PermWash buffer (BD Pharmingen). The intracellular staining was performed using αCD3-APC-Cy7 (clone 145-2C11), αIFN-γ-APC (clone XMG1.2), αIL2-FITC (clone JES6-5H4), αTNFα-PE (clone MP6-XT22) mAbs for 45 minutes on ice. After three more washes with PermWash buffer, cells were resuspended in PBS-FBS, and one million events were acquired in a FACSCanto flow cytometer (BD biosciences), and analysed using FlowJo software (version 9.3, Tree Star, San Carlo, CA). All antibodies used were purchased from BD Pharmingen.

#### CFSE-based proliferation assay

Three hundred thousand splenocytes from immunized mice were assayed for their ability to proliferate *in vitro* using the CFSE dilution based proliferation assay after stimulation with 5 μg/mL of the MSP1_33_ and MSP1_19_ recombinant proteins or with peptides DYDVVYLKPLAGMYK and AKFVAAWTLKAAA, exactly as described in ref. 46.

### Data Analysis

One-way ANOVA followed by Tukey’s honestly significantly different (HSD) test were used to calculate statistical significance (p-values). Prism 5 software (GraphPad Software Inc, LA Jolla, CA) was used for all tests and differences were considered significant when p ≤ 0.05.

## Additional Information

**How to cite this article**: Amorim, K. N. S. *et al*. The presence of T cell epitopes is important for induction of antibody responses against antigens directed to DEC205^+^ dendritic cells. *Sci. Rep.* **6**, 39250; doi: 10.1038/srep39250 (2016).

**Publisher's note:** Springer Nature remains neutral with regard to jurisdictional claims in published maps and institutional affiliations.

## Supplementary Material

Supplementary Information
