# Supplementary material for: The presence of T cell epitopes is important for induction of antibody responses against antigens directed to DEC205+ dendritic cells
Source: Sci Rep. 2016 Dec 21;6:39250. doi: 10.1038/srep39250 (PMC5175286; doi:10.1038/srep39250)
Supplement: Supplementary Information [file srep39250-s1.pdf]

The presence of T cell epitopes is important for induction of antibody responses against antigens directed to DEC205$^+$ dendritic cells

Kelly N. S. Amorim[1], Eline V. Rampazo[1], Renan Antonialli[1], Marcio M. Yamamoto[1], Mauricio M. Rodrigues[2,3], Irene S. Soares[3,4] and Silvia B. Boscardin[1,3*]

[1] Laboratory of Antigen Targeting to Dendritic Cells, Department of Parasitology, Institute of Biomedical Sciences, University of São Paulo, São Paulo, 05508-000, Brazil

[2] CTCMol, Federal University of São Paulo, São Paulo, 04044-010, Brazil

[3] National Institute for Science and Technology in Vaccines, Belo Horizonte, 31270-910, Brazil

[4] Department of Clinical and Toxicological Analysis, School of Pharmaceutical Sciences, University of São Paulo, São Paulo, 05508-900, Brazil

* sbboscardin@usp.br

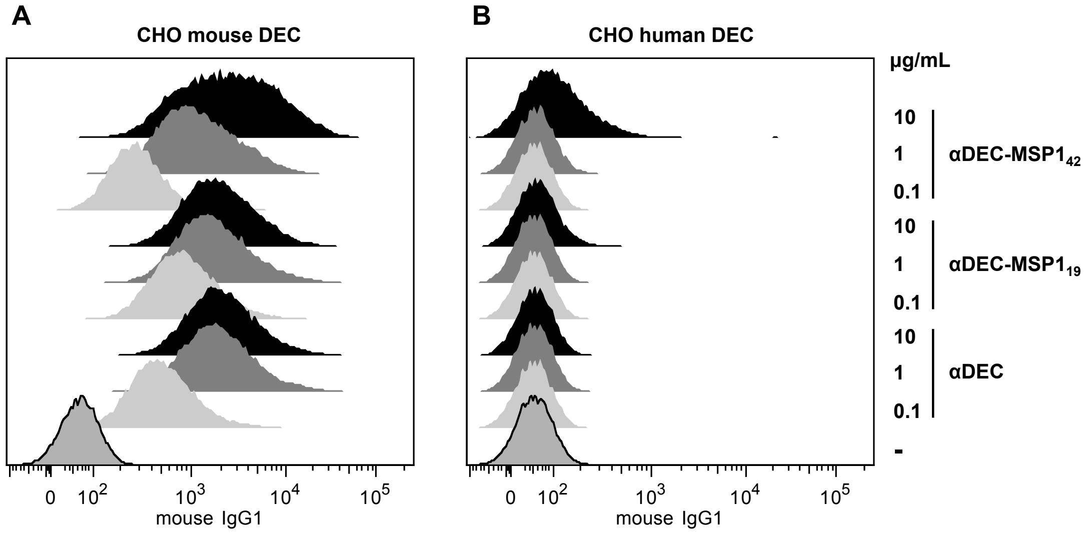

Supplementary Figure 1. Hybrid αDEC-MSP1$_{42}$ and αDEC-MSP1$_{19}$ mAbs retain their ability to bind to the DEC205 receptor expressed on the surface of CHO cells. One hundred thousand CHO cells expressing the murine (A) or the human (B) DEC205 receptor were incubated on ice with 10, 1 or 0.1 µg/mL of the hybrid αDEC-MSP1$_{42}$, αDEC-MSP1$_{19}$ or αDEC mAbs. Binding was detected on 30,000 cells using an anti-mouse IgG1-PE antibody. One experiment representative of three is shown. Analysis was performed using FlowJo software.

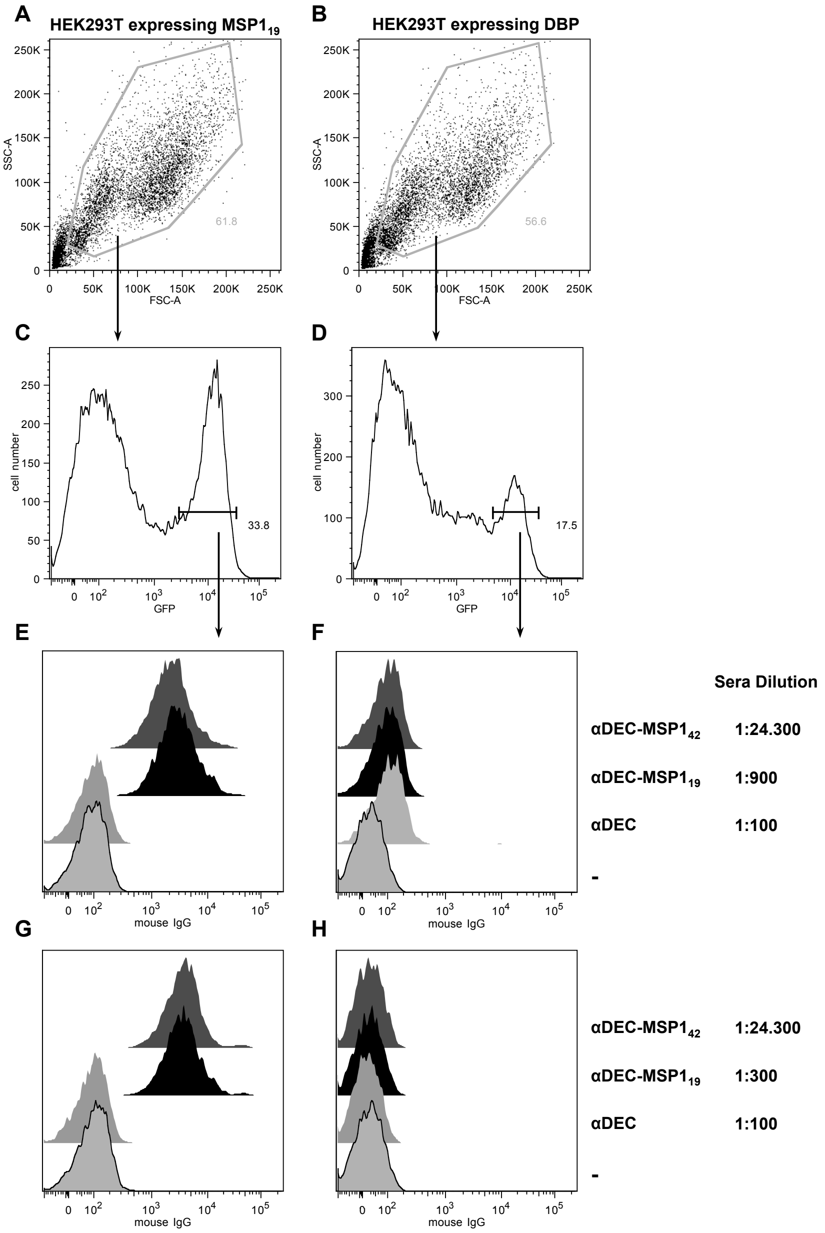

Supplementary Figure 2. Anti-MSP1$_{19}$ antibodies raised in mice immunized with αDEC-MSP1$_{42}$ or αDEC-MSP1$_{19}$ are able to bind to HEK293T cells transiently expressing the MSP1$_{19}$ protein on their surfaces. HEK 293T cells were transiently transfected with plasmids expressing MSP1$_{19}$ (A) or DBP II (B) together with GFP. GFP^{+} cells expressing either MSP1$_{19}$ (C) or DBP II (D) were gated after incubation with different dilutions of sera derived from mice C57BL/6 (E and F) or B10.A (G and H) immunized with αDEC-MSP1$_{42}$, αDEC-MSP1$_{19}$ or αDEC. Binding was detected on 30,000 cells using an anti-mouse IgG1-PE antibody. Analysis was performed using FlowJo software. One experiment representative of two is shown.

A

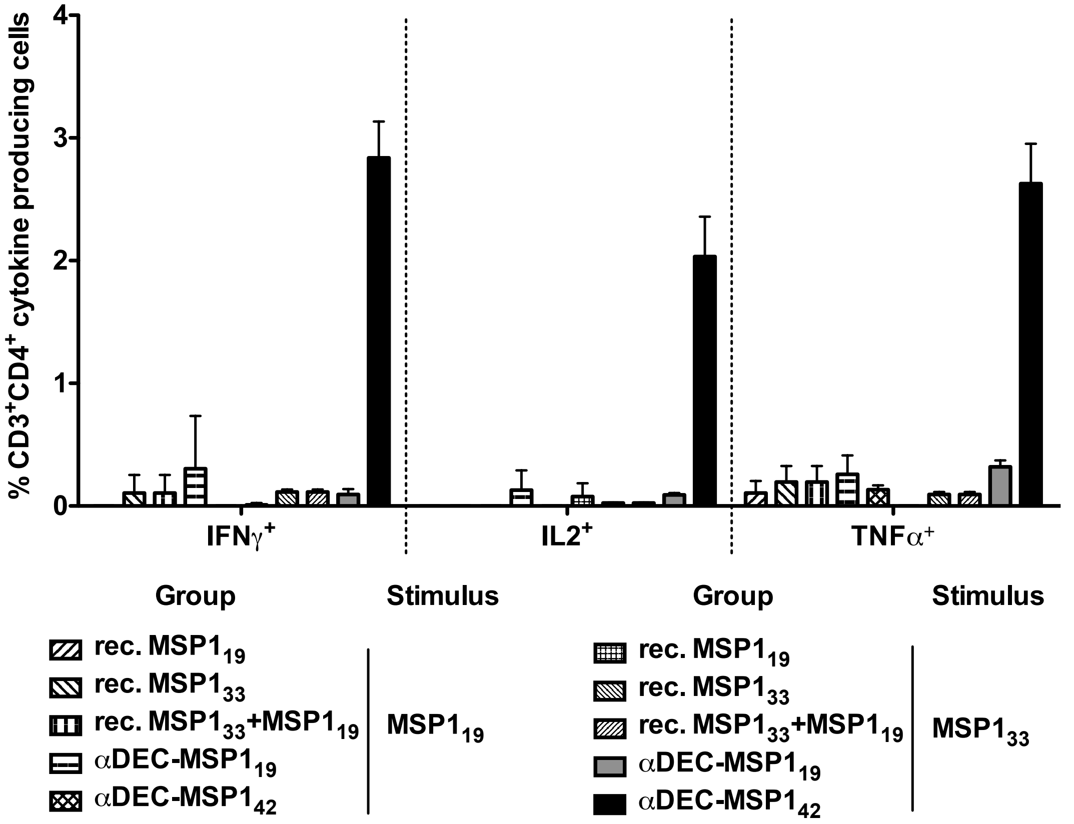
% CD3+CD4+ cytokine producing cells
4
3
2
1
0
IFNγ+
IL2+
TNFα+
Group
Stimulus
rec. MSP1 19
rec. MSP1 33
rec. MSP1 33+MSP1 19
αDEC-MSP1 19
αDEC-MSP1 42
MSP1 19
Group
Stimulus
rec. MSP1 19
rec. MSP1 33
rec. MSP1 33+MSP1 19
αDEC-MSP1 19
αDEC-MSP1 42
MSP1 33

B

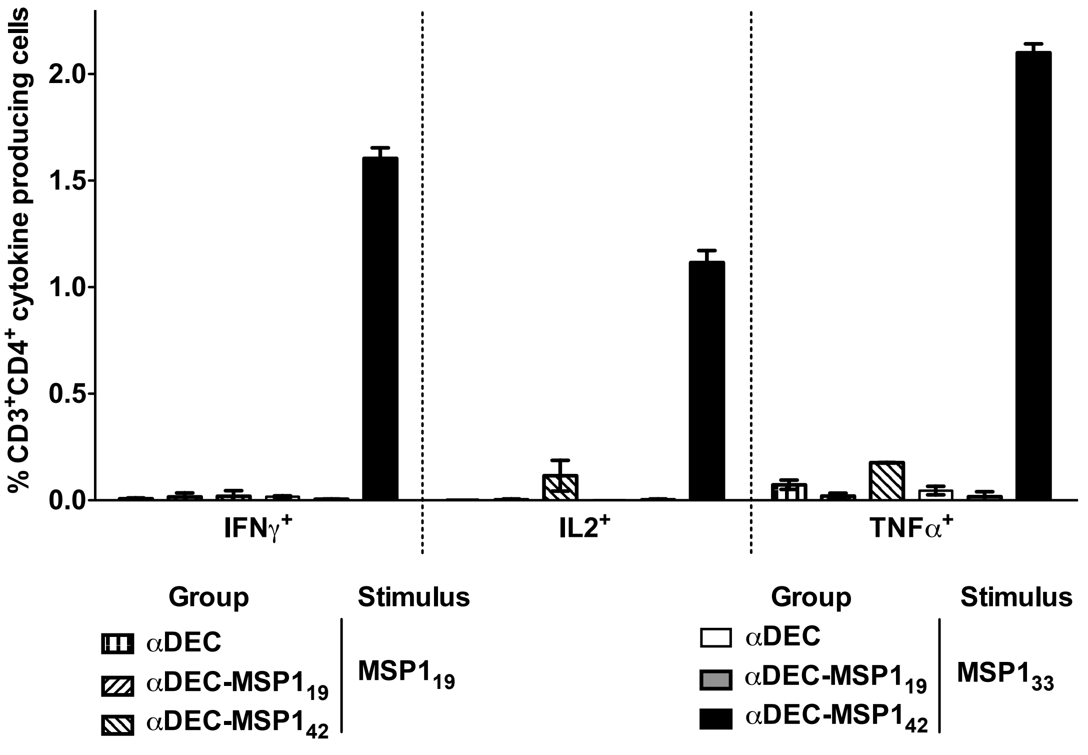
% CD3+CD4+ cytokine producing cells
2.0
1.5
1.0
0.5
0.0
IFNγ+
IL2+
TNFα+
Group
Stimulus
αDEC
αDEC-MSP1 19
αDEC-MSP1 42
MSP1 19
Group
Stimulus
αDEC
αDEC-MSP1 19
αDEC-MSP1 42
MSP1 33

Supplementary Figure 3. Mice immunized with the hybrid αDEC-MSP1$_{42}$ mAb present CD4$^+$ T cells that produce high frequencies of inflammatory cytokines when restimulated with the recombinant MSP1$_{33}$ protein. Groups of mice (n=3) were immunized as described in Figure 2. IFN-γ, IL-2 and TNFα were detected by intracellular staining 20 days after the administration of the booster dose in C57BL/6 (A) and B10.A (B) mice. Total splenocytes were stimulated with 5 μg/ml of MSP1$_{33}$ or MSP1$_{19}$ recombinant proteins. Graphs show the percentage of cells producing IFN-γ, IL-2 and TNFα in the CD3$^+$CD4$^+$ gate after subtracting the values obtained in the absence of any stimulus. The experiment was performed in duplicates using samples from pooled mice. Bars indicate mean ± SD. Results are representative of 2 independent experiments.

Supplementary Materials and Methods

Binding assay do CHO cells expressing DEC205.

The αDEC-MSP1$_{42}$, αDEC-MSP1$_{19}$ or αDEC purified mAbs were diluted to 10, 1, 0.1 µg/ml and incubated with 100,000 CHO cells constitutively expressing the murine DEC205 receptor (CHO mouse DEC205) or the human DEC205 receptor (CHO human DEC205, kindly provided by Dr. Michel Nussenzweig, The Rockefeller University, New York, USA). After a 30-minute incubation on ice, the cells were washed twice with PBS, fetal bovine serum 2% (wash buffer) and incubated with a secondary anti-mouse IgG1-PE antibody (BD biosciences) at a 1:2,000 dilution. The samples were incubated for 40 min on ice and the washed again twice. Thirty thousand events were then read in the FACS Canto (BD biosciences) flow cytometer, and analysed using FlowJo software (version 9.3, Tree Star, San Carlo, CA).

HEK293T transfection and polyclonal antibody binding.

HEK293T cells were transiently transfected as described in [14]. We used 40 µg of plasmids pDE-MSP1$_{19}$ and pDE-DBPII per plate. The empty pDE plasmid was kindly provided by Dr. Joon-Yong Chung (Inje University College of Medicine, South Korea). Two days later, cells were detached using 5 mM EDTA in PBS, washed twice with PBS-FBS, counted and 1x10^5 cells were incubated for 30 min on ice with different dilutions of pooled sera derived from mice immunized with αDEC-MSP1$_{42}$, αDEC-MSP1$_{19}$ or αDEC. The amount of anti-MSP1$_{19}$ present in the sera derived from animals immunized with αDEC-MSP1$_{42}$ or αDEC-MSP1$_{19}$ was normalized by ELISA and two different sera dilutions were used: 1:24,300 for αDEC-MSP1$_{42}$ and 1:900 for αDEC-MSP1$_{19}$. A 1:100 dilution was used for the sera of mice immunized with αDEC. After two additional

washes, cells were incubated with goat anti-mouse IgG-RPE (SouthernBiotech, 1:200) for 20 min on ice. Finally, after two washes, 30.000 events were collected in a FACS Canto flow cytometer (BD biosciences), and analysed using FlowJo software (version 9.3, Tree Star, San Carlo, CA). GFP^+ cells were gated to analyse the binding.
